# Role of the Chemokine Receptors CCR1, CCR2 and CCR4 in the Pathogenesis of Experimental Dengue Infection in Mice

**DOI:** 10.1371/journal.pone.0015680

**Published:** 2010-12-29

**Authors:** Rodrigo Guabiraba, Rafael Elias Marques, Anne-Gaëlle Besnard, Caio T. Fagundes, Danielle G. Souza, Bernhard Ryffel, Mauro M. Teixeira

**Affiliations:** 1 Immunopharmacology, Departamento de Bioquímica e Imunologia, Instituto de Ciências Biológicas, Universidade Federal de Minas Gerais, Belo Horizonte, Minas Gerais, Brazil; 2 Université d'Orléans and CNRS, UMR 6218, Molecular Immunology and Embryology, Orléans, France; 3 Departamento de Microbiologia, Instituto de Ciências Biológicas, Universidade Federal de Minas Gerais, Belo Horizonte, Minas Gerais, Brazil; Federal University of São Paulo, Brazil

## Abstract

*Dengue virus* (DENV), a mosquito-borne flavivirus, is a public health problem in many tropical countries. Recent clinical data have shown an association between levels of different chemokines in plasma and severity of dengue. We evaluated the role of CC chemokine receptors CCR1, CCR2 and CCR4 in an experimental model of DENV-2 infection in mice. Infection of mice induced evident clinical disease and tissue damage, including thrombocytopenia, hemoconcentration, lymphopenia, increased levels of transaminases and pro-inflammatory cytokines, and lethality in WT mice. Importantly, infected WT mice presented increased levels of chemokines CCL2/JE, CCL3/MIP-1α and CCL5/RANTES in spleen and liver. CCR1^-/-^ mice had a mild phenotype with disease presentation and lethality similar to those of WT mice. In CCR2^-/-^ mice, lethality, liver damage, levels of IL-6 and IFN-γ, and leukocyte activation were attenuated. However, thrombocytopenia, hemoconcentration and systemic TNF-α levels were similar to infected WT mice. Infection enhanced levels of CCL17/TARC, a CCR4 ligand. In CCR4^-/-^ mice, lethality, tissue injury and systemic inflammation were markedly decreased. Despite differences in disease presentation in CCR-deficient mice, there was no significant difference in viral load. In conclusion, activation of chemokine receptors has discrete roles in the pathogenesis of dengue infection. These studies suggest that the chemokine storm that follows severe primary dengue infection associates mostly to development of disease rather than protection.

## Introduction

Dengue fever (DF) and its severe forms, dengue hemorrhagic fever (DHF) and dengue shock syndrome (DSS), are mosquito-borne diseases caused by one of four serotypes of *Dengue virus* (DENV 1-4) [1], [2], [3]. DENV is a single-stranded RNA virus that belongs to the *Flaviviridae* family and is transmitted to humans by Aedes mosquitoes [1], [4]. They constitute a serious public health problem in tropical and subtropical areas, where the incidence, distribution and clinical severity of dengue cases have dramatically increased in the last 60 years [4]. Treatment of DF or DHF/DSS is largely supportive and the lack of clinical or laboratory markers for an efficient diagnostic associated to the lack of a vaccine or specific treatment put a serious burden on health systems of low income countries [1], [4], [5].

The pathogenesis of DENV remains poorly understood and involves a complex interplay of viral and host factors, including viral serotype [6], [7], genotype [6], and the genetic background of the host [8], [9]. Secondary infection by a heterologous serotype has been shown to be the single greatest risk factor for DHF/DSS in human subjects [9], [10], [11], [12] although severe disease in primary infections has also been also reported [6], [13], [14], [15].

DHF/DSS is characterized by hemorrhagic manifestations, thrombocytopenia and hemoconcentration [1], [4], [7], where the dysfunction of vascular endothelial cells that leads to plasma leakage is mediated by host immune response [5], [12], [13]. DENV can interact with immune cells such as dendritic cells (DCs), monocytes/macrophages, hepatocytes and endothelial cells [7], [16], [17], [18], [19], [20], resulting in the production of immune mediators that shape innate and acquired immune responses. High levels of pro-inflammatory cytokines and chemokines, including TNF-α, IL-6, IL-8, CCL2/MCP-1 and IFN-γ, have been reported in patients with severe dengue disease [2], [21], [22], [23]. However, it is not clearly understood how this massive cytokine production is induced and eventually controlled, a phenomenon that also occurs in bacterial sepsis and other shock related syndromes [24], [25].

Chemokines are members of a structurally related family of cytokines involved in leukocyte traffic during inflammation. They are classified according to the relative position of conserved N-terminal cysteine residues, in which CC chemokines represent the most abundant family and have the first 2 cysteines placed adjacently [26], [27]. Chemokine receptors are expressed on the surface of leukocytes and are G protein-coupled receptors containing 7 transmembrane domains [26], [28], [29]. They may also contribute to angiogenesis, recruitment and transmigration of leukocytes, vascular and tissue remodeling, chronification of inflammation, among others [27], [28], [30], [31], [32]. Experimental and epidemiological evidences suggest an important role for chemokines, especially those from the CC family, and their receptors in infectious diseases such as HIV, HSV-1 and other viral infections [27], [33], [34], [35], [36].

Recent clinical studies in endemic areas describe a correlation between dengue disease outcome and levels of CC chemokines, including CCL4/MIP1-β and CCL3/MIP1-α, both ligands for the CCR1 receptor, and for CCL2/MCP-1, the ligand for CCR2 [37], [38], [39]. A link between CCL5/RANTES, a CCR1/CCR5 ligand, and hepatic dysfunction had already been shown [40], [41]. In addition, CCL2/MCP-1 and IL-8 are intimately related to hypotension, thrombocytopenia and hemorrhagic shock [21], [22], [37], [41], [42]. However, the relevance of chemokines for the pathogenesis and host response in the context of dengue infection remains to be determined.

We have recently developed a model of dengue infection in mice that resembles many of the features of severe dengue infection in humans, including thrombocytopenia, increased vascular permeability, cytokine storm, systemic inflammation and death [43], [44], [45]. Using this model, we have investigated the role of CC chemokine receptors CCR1, CCR2 and CCR4 during experimental DENV infection. Ligands to these receptors have been shown to be elevated in human and experimental dengue infection [21], [37], [38], [39], [40], [41], [46], [47].

## Materials and Methods

### Ethics statement

All experimental procedures were approved and complied with the French government's ethical and animal experiment regulations and the Comité National de Réflexion Ethique sur l'Expérimentation Animale, CNRS, Orléans, France (CLE CCO 2009-013).

### Mice

Eight to ten week-old male wild type (WT) C57BL/6 (H-2D^b^) mice were purchased from Janvier (Le Genest-St-Isle, France). CCR1^–/–^, CCR2^–/–^ and CCR4^–/–^ male mice, eight to ten week-old, backcrossed at least 10 times in C57BL/6, were bred in the animal facility of the Transgenose Institute (CNRS, Orléans, France). All animals were kept under controlled temperature (23°C) with a strict 12 h light/dark cycle, autoclaved food and water available *ad libitum* under SPF (specific pathogen–free conditions).

### Virus

The mouse-adapted DENV serotype 2 strain (DENV-2) P23085 was obtained from the State Collection of Viruses, Moscow, Russia, and adapted as previously described [44]. Sequence of portions of E and NS1 genes of the adapted virus was deposited previously at GenBank under the accession number AY927231. Virus adaptation was performed in a maximum containment biosafety level-3 (BSL-3) of the SRC VB “Vector”, Koltsovo, Russia. For the current set of experiments, the last 2 passages of the mouse-adopted DENV-2 strain was performed in LLC-MK2 cells (Kidney, Rhesus monkey, ATCC) to produce stocks which were stored in Dulbecco's Modified Eagles Medium (DMEM, Sigma-Aldrich) at −80°C. To calculate virus titers in LLC MK2 cells supernatants, expressed as LD_50_, groups of ten mice were inoculated i.p. with serial dilutions of the virus and lethality recorded. The titer of our DENV-2 stock was 10^5^ LD_50_/ml or 2×10^6^ PFU/ml of LLC MK2 supernatant, as calculated in 4-week-old BALB/c mice, a more susceptible lineage [48].

### Infection and experimental design

For DENV infection, mice were handled and kept in a biosafety level 3 (BSL-3) in the animal facility of the Transgenose Institute (CNRS, Orléans, France). For the evaluation of lethality and inflammation, mice were inoculated i.p. with DENV-2 virus (10 LD_50_) diluted in 100 µl of endotoxin-free DPBS (Gibco). One LD_50_ corresponds to the inoculum necessary to kill 50% of 4 weeks old BALB/c mice and correspond to approximately 20 PFU, as assessed in LLC-MK2 cells. Lethality rates and body weight loss were evaluated every 12 h until day 14 post infection (p.i.). The other parameters were evaluated at day 6 after i.p. inoculation of the virus, a time point where animals were still alive and showed significant clinical signs of disease. In all experiments using genetically deficient mice (KO mice), experiments with the relevant WT controls were performed in parallel. Viral stocks were prepared in LLC MK2 cells and non-infected animals were inoculated with DMEM supernatants from non-infected LLC MK2 cells diluted in a similar manner. At day 6 p.i., mice were anesthetized i.p. with a ketamine (100 mg/kg)/xylazine (10 mg/kg) solution diluted in sterile DPBS and blood were recovered for serum preparation and hematological analysis. Then, mice were killed by cervical displacement and spleen and liver samples were recovered for cytokine dosage, FACS analysis and/or viral titration. Samples were stocked at −80°C prior to the analysis. Liver samples were also used for histological analysis.

### Titration of virus

Mice were assayed for viral titers in the liver, as previously described [43], [45]. Briefly, tissue samples were prepared as 10% (w/v) homogenates in DMEM without fetal bovine serum (FBS). Viral load in the supernatants of tissue homogenates was assessed by direct plaque assays using LLC-MK2 cells. Samples of organ homogenates were diluted serially and placed in duplicate into 6-wells plates (TPP, Techno Plastic Products AG) of LLC-MK2 cell monolayers and incubated for 1 h. An overlay solution containing 199 medium (Gibco) with Earle's salts, L-glutamine and 3% FBS in 1,5% CMC (Carboxymethylcellulose, Sigma) was added to each well and the cultures were incubated for 9 days. Cultures were stained with crystal violet for enumeration of viral plaques. The results were measured as plaque forming units (PFU) per 100 mg of tissue weight. The limit of detection of the assay was 10 PFU/100 mg of tissue.

### Evaluation of blood parameters

Blood was obtained from the brachial plexus of anesthetized mice in heparin-containing syringes, at the indicated times, and stocked in heparinized tubes prior to analysis. The final concentration of heparin was 50 U/ml. Platelets, hematocrit and lymphocytes were evaluated in a Coulter Counter (S-Plus Jr, Beckman Coulter). Results are presented as percentage of hematocrit and lymphocytes, and platelets per µl of blood.

### AST and ALT dosage

Aspartate aminotransferase (AST) and Alanine aminotransferase (ALT) were dosed in non-hemolyzed serum samples as marker enzymes associated to hepatic damage due to DENV-2 infection. The colorimetric assays to evaluate the aforementioned enzymes were conducted following the manufacturer's protocol (Quibasa, Bioclin, Brazil).

### Determination of Myeloperoxidase (MPO) activity

For MPO analysis, as an indirect index of neutrophil accumulation, spleen and liver homogenates were prepared in 1 ml of PBS containing 0.5% hexadecyltrimethyl ammonium bromide (HTAB) and 5 mM EDTA using a Dispomix tissue homogenizer (Medic Tools) and the protocol was followed as already described [49]. Results are expressed as arbitrary units (OD 492 nm) and were corrected for the activity of other peroxidases, which were not inhibited by 3-amino-1,2,4-triazole.

### Quantification of cytokines and chemokines concentrations

The concentrations of TNF-α, IFN-γ, IL-6, CCL17/TARC, CCL2/JE, CCL3/MIP-1α and CCL5/RANTES in serum or tissue samples were measured by ELISA using commercially available antibodies and according to the procedures supplied by the manufacturer (R&D Systems, Minneapolis). ELISA measurements for a given experiment were conducted in the same plate. Results are expressed as pg/ml or pg per 100 mg of tissue. The detection limit of the ELISA assays was in the range of 4–8 pg/ml.

### Histopathology

A portion of liver was obtained from mice at day 6, immediately fixed in 4% buffered formalin and tissues fragments were embedded in paraffin. Tissue sections (4 µm thick) were stained with hematoxylin and eosin (H&E) and examined under light microscopy**.** Pictures were taken using the QCapture Pro 6.0 software (QImaging, Canada).

### Flow cytometry

Spleens were collected, homogenized in sterile strainers and resuspended in PBS 2% FBS. Red blood cells were removed with lysis buffer (Sigma-Aldrich). Cells were stained for extracellular molecular expression patterns using monoclonal antibodies (mAb) against mouse CD3 (PerCP-Cy5-conjugated), DX5 (FITC-conjugated), CD4 (Pacific Blue-conjugated), CD8 (APC-Cy7-conjugated), F4/80 (PE-conjugated) CD69 (PE-conjugated), CD11b (PerCp-Cy5.5-conjugated), Ly6G (PE-Cy7-conjugated), CD86 (APC-conjugated) and isotype controls. All antibodies were purchased from BD Pharmingen (Le Pont de Claix, France). In all cases, 5×10^5^ to 1×10^6^ gated events were acquired for later analysis. The frequency of positive cells was analyzed using a gate that included lymphocytes, granulocytes and monocytes/macrophages. Limits for the quadrant markers were always set based on negative populations and isotype controls. Cells were acquired on a BD FACSCanto II (BD Biosciences) cytometer and analyzed using the FlowJo 7.5.3 software (TreeStar Inc.). Analysis in FlowJo software took into account size and granularity of populations. Frequency in number of an analyzed population in front of total acquired events was used in the construction of graphs.

### Statistical analysis

Results are shown as means ± S.E.M. Differences were compared by using analysis of variance (ANOVA) followed by Student-Newman-Keuls post-hoc analysis. Differences between lethality curves were calculated using Log rank test (Graph Prism Software 4.0). Results with a P<0.05 were considered significant. All data are representative of at least 2 experiments (n = 5–12 mice).

## Results

### Lethality rate after DENV-2 infection in mice

**Figure 1:**
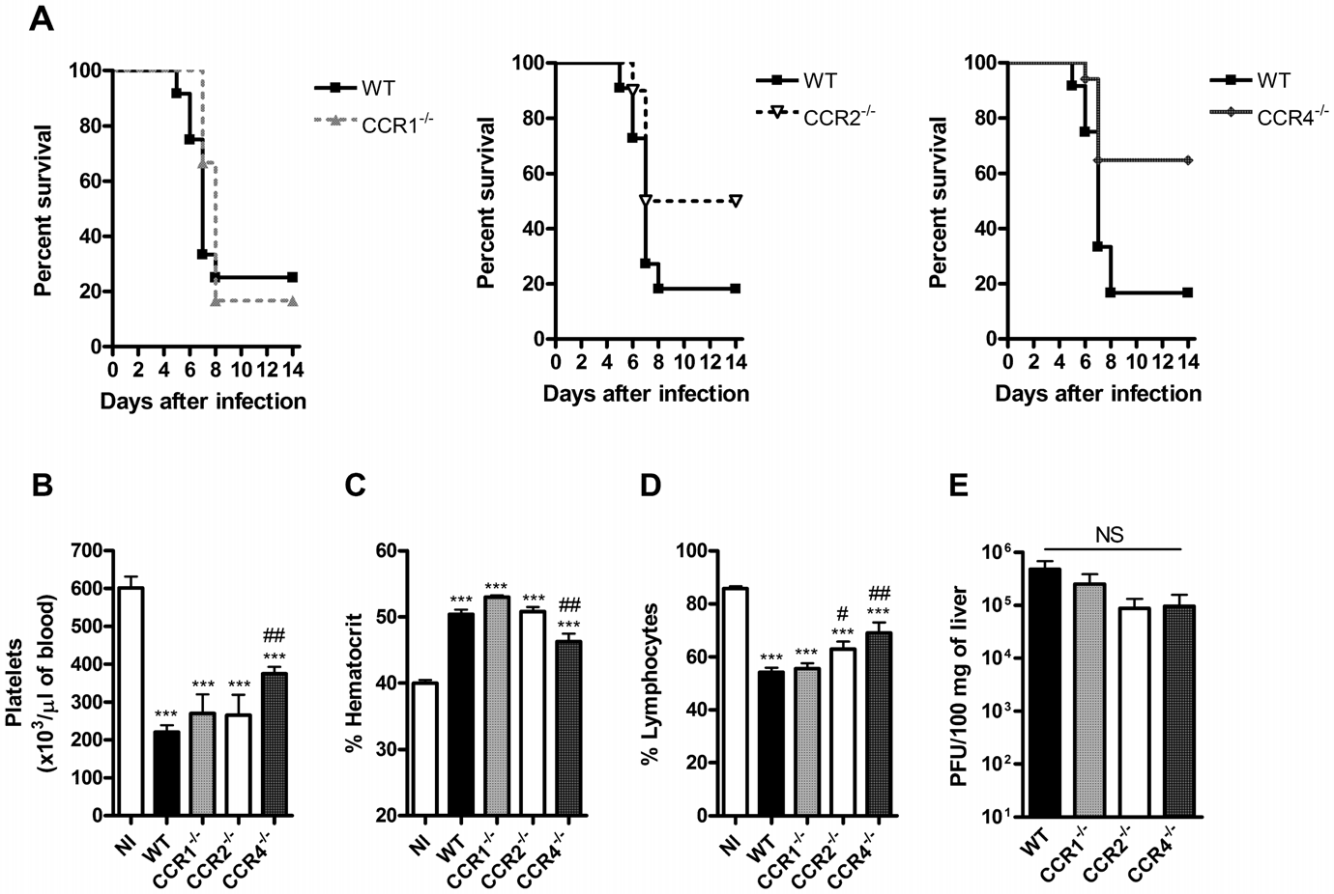
Lethality rate, hematological alterations and viral load upon DENV-2 infection. WT or CCR1^–/–^, CCR2^–/–^ and CCR4^–/–^ (KO) mice were infected i.p. with 10 LD_50_ of DENV-2 and then monitored for lethality until day 14. In panel A, percentages of survival (n = 10–12). Hematological analysis were done at day 6 p.i. for changes in platelets count (B) hematocrit (C) and lymphocytes (D) in the blood of non-infected and infected-WT and KO mice. In panel E, viral loads recovered from the liver of WT and KO mice at day 6 p.i. shown as the log of PFU/100 mg of tissue. Results are expressed as mean ± SEM and are representative of at least two experiments (n = 5–12 mice). *** P<0.001 when compared to non-infected mice. # P<0.05, ## P<0.01 when compared to WT infected mice. NI: non-infected mice. NS: Not significant.

Mice infected with a mouse-adapted DENV-2 strain have a clinical presentation that resembles DHF/DSS in humans, including thrombocytopenia and increased vascular permeability that eventually leads to shock and death [43], [44], [45]. As seen in Figure 1A, infection of WT mice with an inoculum of 10 LD_50_ killed approximately 80% of mice around days 6 to 8 after inoculation. Lethality rate in CCR1^–/–^ mice was similar to that of WT mice. In contrast, there was significant protection of infected CCR2^–/–^ and CCR4^–/–^ mice (P = 0.0312 and P = 0.0091, respectively). Indeed, approximately 55% and 65% of CCR2^–/–^ and CCR4^–/–^ mice, respectively, were still alive till day 14 after inoculation. Infection of WT mice was associated with rapid weight loss starting at day 4 p.i. and leading to loss of about 5% at day 7 for surviving animals. Of notice, CCR1^–/–^ mice lost weight in a manner similar to that of WT animals and weight loss was significantly decreased in CCR2^–/–^ and CCR4^–/–^ mice at about 3% (data not shown).

### Hematological parameters and viral load

Thrombocytopenia is a common finding in patients with dengue fever and DHF/DSS, but does not appear to correlate with disease severity or outcome [1], [7]. Thrombocytopenia is first observed at day 3 and peak at days 6-7 after infection [6], [37], [50]. In the present study, platelet levels were evaluated at day 6 p.i, a time point at which levels were lowest and the percentage of surviving animals maximal. Platelet number in infected WT mice was about 30% of non-infected animals (Figure 1B). There was a similar fall in platelet number in both CCR1^–/–^ and CCR2^–/–^ infected mice. The decrease of platelet numbers in CCR4^–/–^mice was slightly less than in WT mice (Figure 1B).

Plasma leakage and consequent hemoconcentration is a relatively late event and a major finding in patients with severe dengue [21], [50], [51], [52], [53]. Infection of WT mice induced significant increase in hemoconcentration (Figure 1C). Similar increase was observed in infected CCR1^–/–^ and CCR2^–/–^ deficient mice. Hemoconcentration occurred but was of lower magnitude in infected CCR4^–/–^ mice than in their WT controls (Figure 1C).

Lymphopenia is a common phenomenon associated to severe manifestations of dengue in humans [50], [51]. As seen in Figure 1D, there was marked reduction in percentage of blood lymphocytes in WT mice upon DENV-2 infection when compared to non-infected animals. Although no differences were observed between WT and CCR1^–/–^-infected mice, CCR2^–/–^ and CCR4^–/–^ mice had reduced blood lymphopenia when compared to WT-infected mice.

Despite differences observed in lethality and hematology, there were no significant differences in viral titers in liver of WT and CCR1, CCR2 and CCR4 KO mice (Figure 1E).

Of interest, no alterations in haematological parameters, such as thrombocytopenia and hemoconcentration, and viral load, were observed at day 14 in mice which survived till this timepoint (data not shown).

### Liver inflammation and injury

**Figure 2:**
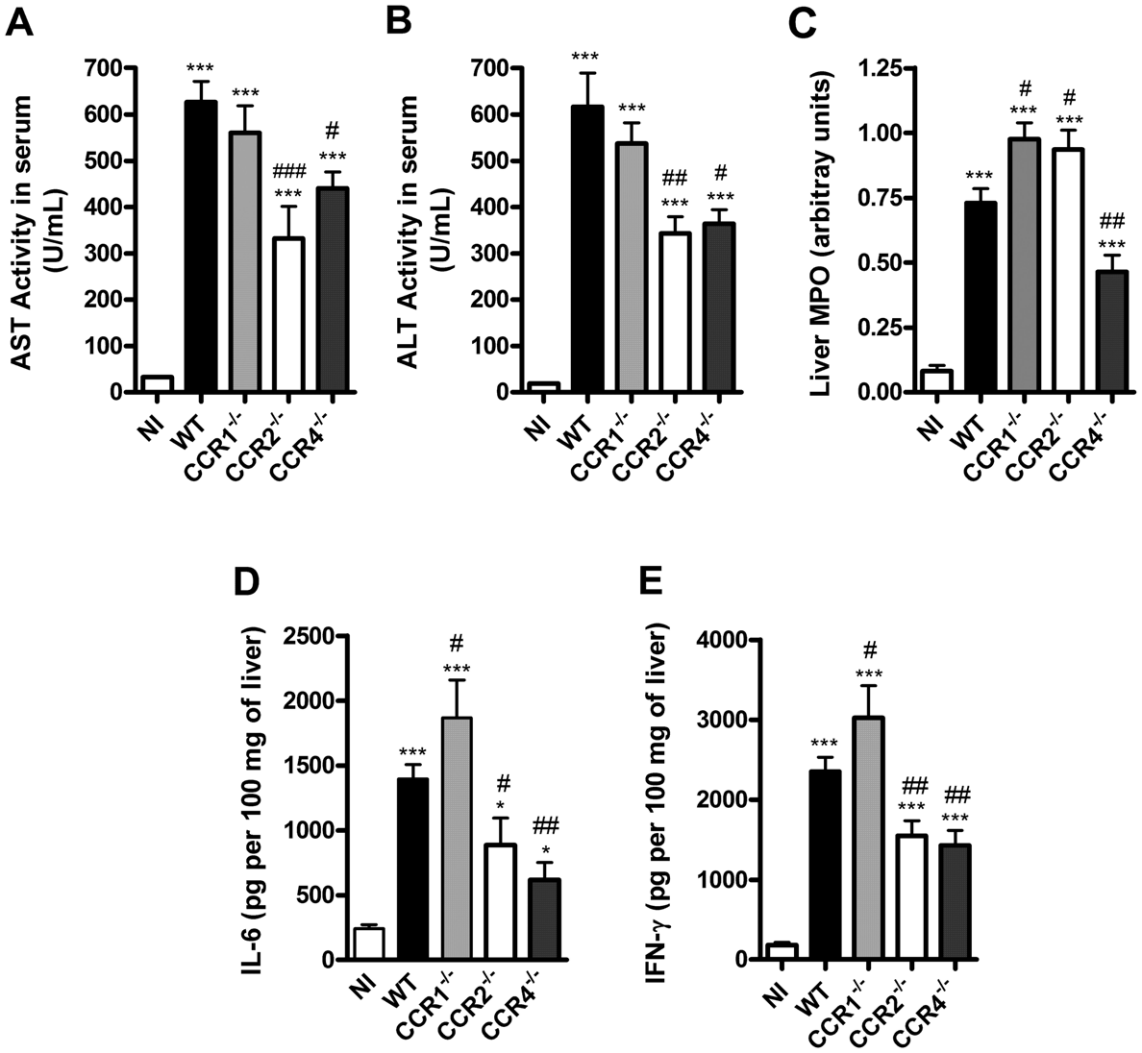
Liver inflammation and injury upon DENV-2 infection. WT or KO mice were infected i.p. with 10 LD_50_ of DENV-2 and then sacrificed at day 6 for blood and tissue samples. AST (A) and ALT (B) were dosed in serum of WT and KO mice as markers of hepatic injury. MPO activity, as an index of neutrophil accumulation, was evaluated in liver (C). Concentrations of cytokines IL-6 (D) and IFN-γ (E) were evaluated in liver homogenates by ELISA and are expressed as pg per 100 mg of tissue. Results are expressed as mean ± SEM and are representative of at least two experiments (n = 5–6). * P<0.05, *** P<0.001 when compared to non-infected mice. # P<0.05, ## P<0.01, ### P<0.001 when compared to WT infected mice. NI: non-infected mice.

**Figure 3:**
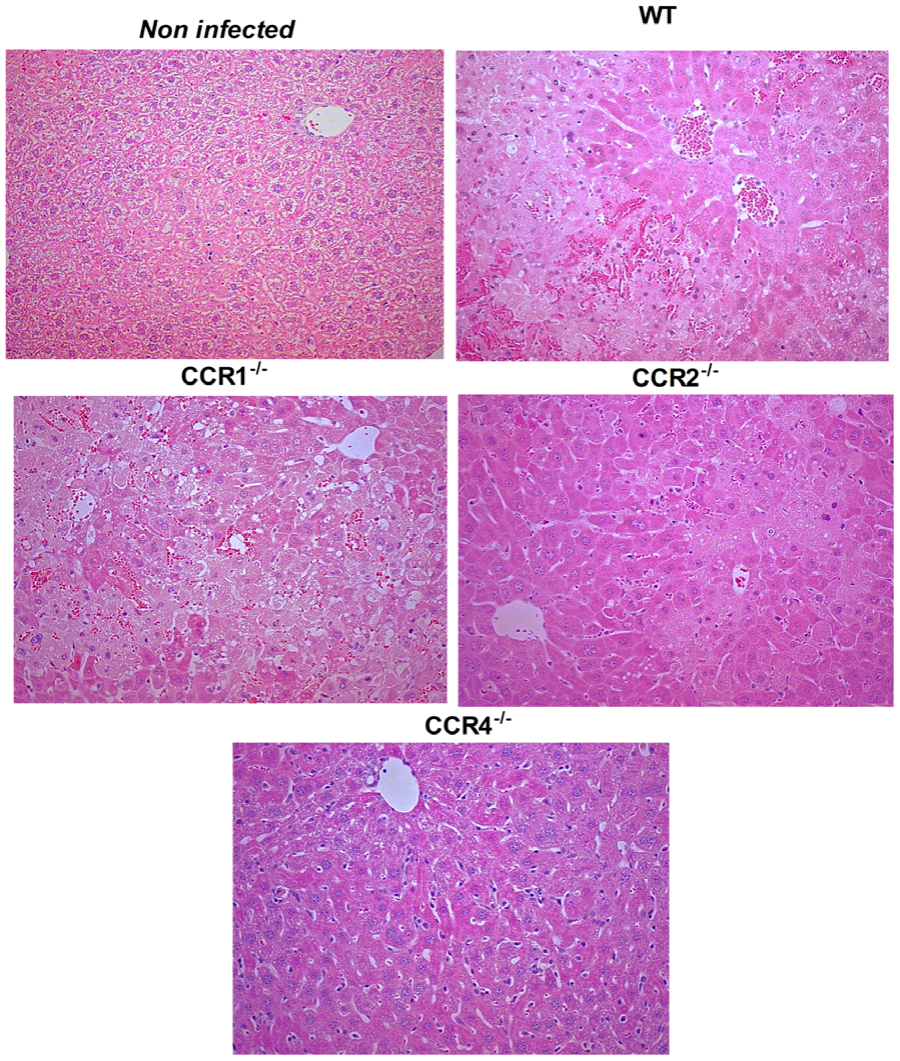
Histological changes in liver upon DENV-2 infection in mice. WT or KO mice were infected i.p. with 10 LD_50_ of DENV-2 and then sacrificed at day 6 for tissue samples. Hematoxylin & Eosin stained liver sections from non-infected and DENV-2 infected WT, CCR1^–/–^, CCR2^–/–^ and CCR4^–/–^ mice, showing different degrees of congestion, hemorrhage, hepatocyte degeneration and necrosis. Each slide presented in the panel is representative of at least 10 different fields (n = 5–6 mice). Magnification: 400X.

The liver is a major target organ in severe cases of dengue infection [7], [16], [41]. Infection of mice with DENV-2 resulted in significant increase in serum levels of the transaminases AST and ALT at day 6 (Figure 2A and B). Granulocytes often become activated, accumulate in tissues and may contribute to organ damage in the context of DENV infection [54]. There was significant accumulation of neutrophils in the liver (Figure 2C) and spleen (data not shown) of infected WT mice, as assessed by tissue levels of MPO. The changes above were accompanied by an increase in liver concentration of IL-6 and IFN-γ (Figure 2D and E). Liver sections of infected WT revealed signs of congestion, haemorrhage, hepatocyte degeneration and necrosis (Figure 3).

Overall, CCR1^–/–^ mice had similar degree of damage as WT mice, with only slightly enhanced MPO activity and levels of both IL-6 and IFN-γ in liver (Figure 2). MPO levels were also slightly increased in liver of CCR2^–/–^ mice, but there was decreased liver injury, as seen by decreased levels of transaminases in serum and by histology. Decreased liver damage was associated with decreased local production of both IL-6 and IFN-γ (Figure 2). Systemic levels of transaminases and local levels of MPO and cytokines were significantly decreased in infected CCR4^–/–^ mice, a finding consistent with the amelioration of liver damage as demonstrated in tissues sections (Figure 3).

**Figure 4:**
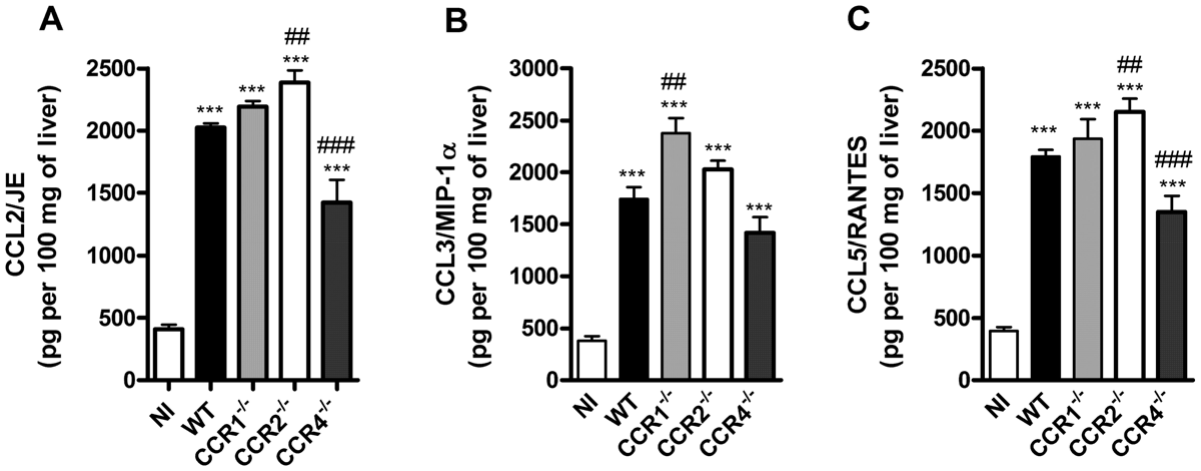
Chemokine production in liver upon DENV-2 infection in mice. WT or KO mice were infected i.p. with 10LD_50_ of DENV-2 and then sacrificed at day 6 for tissue samples. CCL2/JE (A), CCL3/MIP-1α (B) and CCL5/RANTES (C) were evaluated in liver homogenates by ELISA and are expressed as pg per 100 mg of tissue. Results are expressed as mean ± SEM and are representative of at least two experiments (n = 5–6 mice). *P<0.05, *** P<0.001 when compared to non-infected mice. # P<0.05, ## P<0.01, ### P<0.001 when compared to WT infected mice. NI: non-infected mice.

Levels of the chemokines CCL2/JE, CCL3/MIP-1α, CCL5/RANTES and CCL17/TARC were evaluated in liver samples of infected WT and chemokine receptor-deficient mice. With the exception of CCL17, which did not rise above basal levels in the liver (data not shown), there was significant increase of CCL2, CCL3 and CCL5 after DENV-2 infection (Figure 4A–C). In infected CCR1^–/–^ mice, levels of CCL3 increased slightly above levels found in WT mice. CCR2^–/–^ mice showed slightly enhanced levels of CCL2 and CCL5, a finding consistent with the enhanced MPO levels observed in these mice. Levels of CCL2 and CCL5 were decreased in liver samples of CCR4^–/–^ mice (Figure 4A and C). There were no differences in basal levels of cytokines/chemokines between both non-infected WT and KO mice (data not shown).

### Systemic cytokine and chemokine response

**Figure 5:**
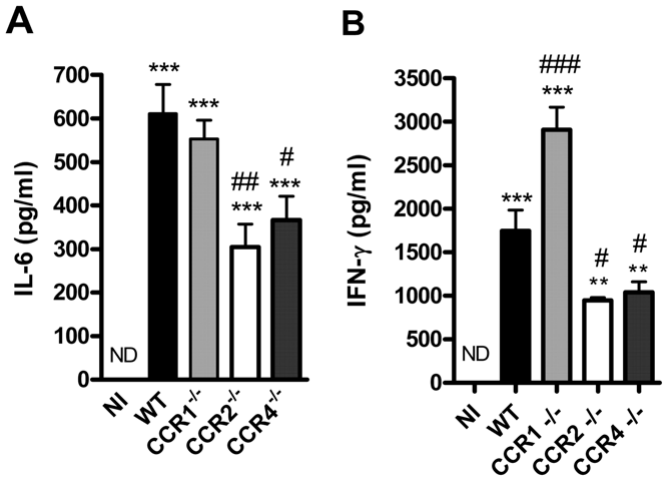
Cytokine production in serum upon DENV-2 infection in mice. WT or KO mice were infected i.p. with 10 LD_50_ of DENV-2 and then sacrificed at day 6 for blood samples. IL-6 (A) and IFN-γ (B) were evaluated in serum by ELISA and are expressed as pg/ml. Results are expressed as mean ± SEM and are representative of at least two experiments (n = 5–6 mice). ** P<0.01, *** P<0.001 when compared to non-infected mice. # P<0.05, ## P<0.01, ### P<0.001 when compared to WT infected mice. NI: non-infected mice.

Previous studies have shown that IL-6 and IFN-γ are elevated systemically in patients with dengue or in experimental models of the infection [37], [41], [44], [47], [55], [56], [57]. Indeed, very large concentrations of both IL-6 and IFN-γ were detectable in serum of DENV-2-infected WT mice (Figure 5A and B). In CCR1^–/–^ mice, concentration of IFN-γ was similar to WT mice and there was an increase in serum levels of IL-6. Levels of both cytokines were decreased in serum samples of CCR2^–/–^ and CCR4^–/–^ mice, as compared to infected WT mice (Figure 5).

**Figure 6:**
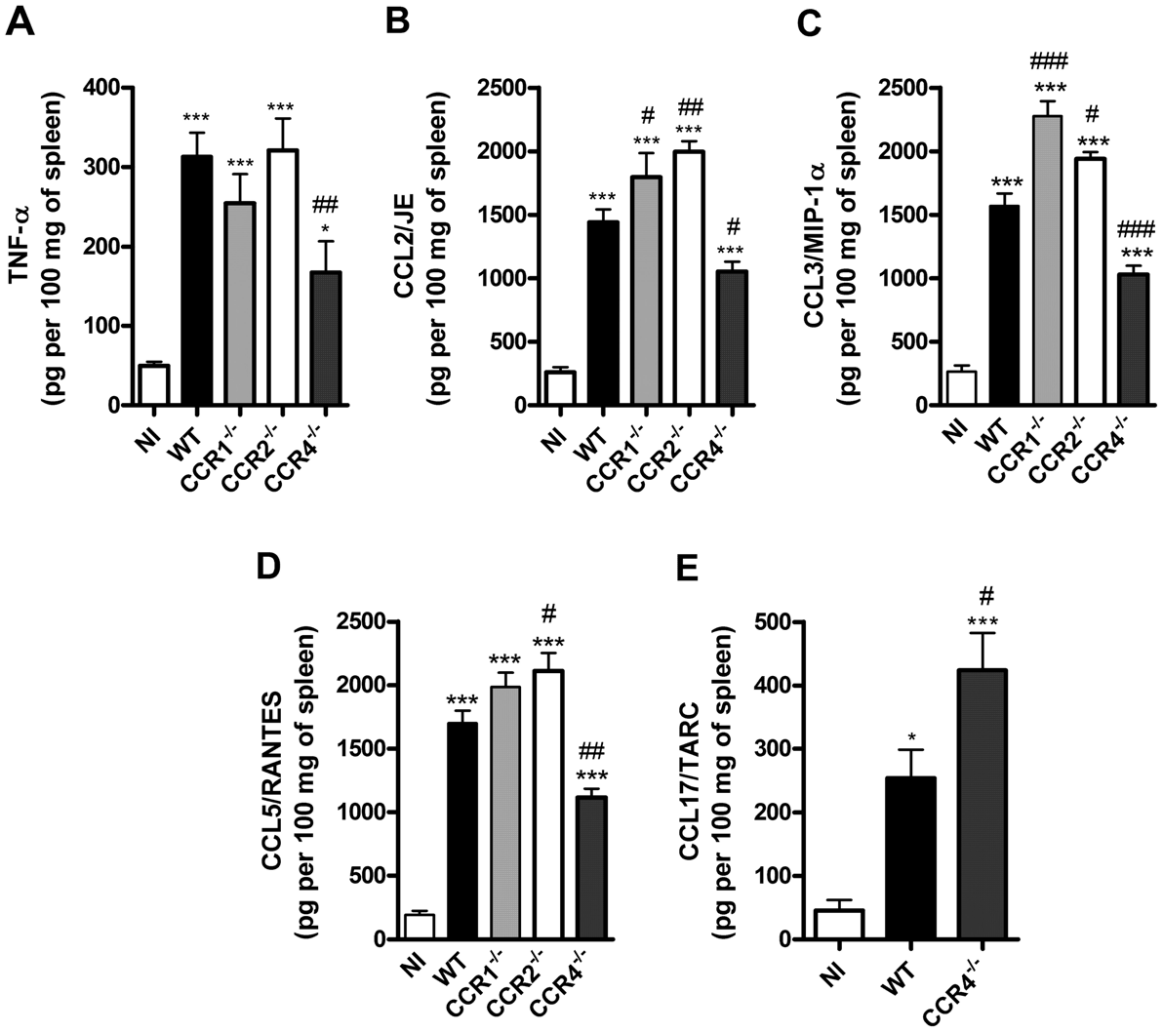
Cytokine and chemokine production in spleen upon DENV-2 infection in mice. WT or KO mice were infected i.p. with 10 LD_50_ of DENV-2 and then sacrificed at day 6 for tissue samples. TNF-α (A), CCL2/JE (B), CCL3/MIP-1α (C), CCL5/RANTES (D) and CCL17/TARC (E) were evaluated in spleen homogenates by ELISA and are expressed as pg per 100 mg of tissue. Results are expressed as mean ± SEM and are representative of at least two experiments (n = 5–6 mice). * P<0.05, *** P<0.001 when compared to non-infected mice. # P<0.05, ## P<0.01, ### P<0.001 when compared to WT infected mice. NI: non-infected mice.

Levels of TNF-α and chemokines were measured in spleen homogenates to get a glimpse of the systemic inflammatory response to dengue infection and because available serum samples were used for transaminases and IL-6/IFN-γ determinations. As seen in Figure 6A, levels of TNF-α were significantly enhanced after infection of WT mice. Similarly, there was marked increase in levels of CCL2, CCL3, CCL5 and CCL17 in spleen homogenates of infected WT mice (Figure 6B-E). Levels of all chemokines and TNF-α were decreased in infected CCR4^–/–^ mice when compared to WT mice, with the exception of CCL17 (Figure 6E). Indeed, the levels of CCL17, which binds to CCR4, were greatly enhanced in CCR4^–/–^ mice. Levels of TNF-α were similar in infected CCR1^–/–^ and CCR2^–/–^ mice when compared to WT mice. Levels of CCL3 are enhanced in CCR1^–/–^ mice and levels of CCL2 and CCL5 in spleen homogenates of infected CCR2^–/–^ mice (Figure 6). There were no differences in basal levels of cytokines/chemokines between both non-infected WT and KO mice (data not shown).

### Number and activation of leukocytes in spleen

**Figure 7:**
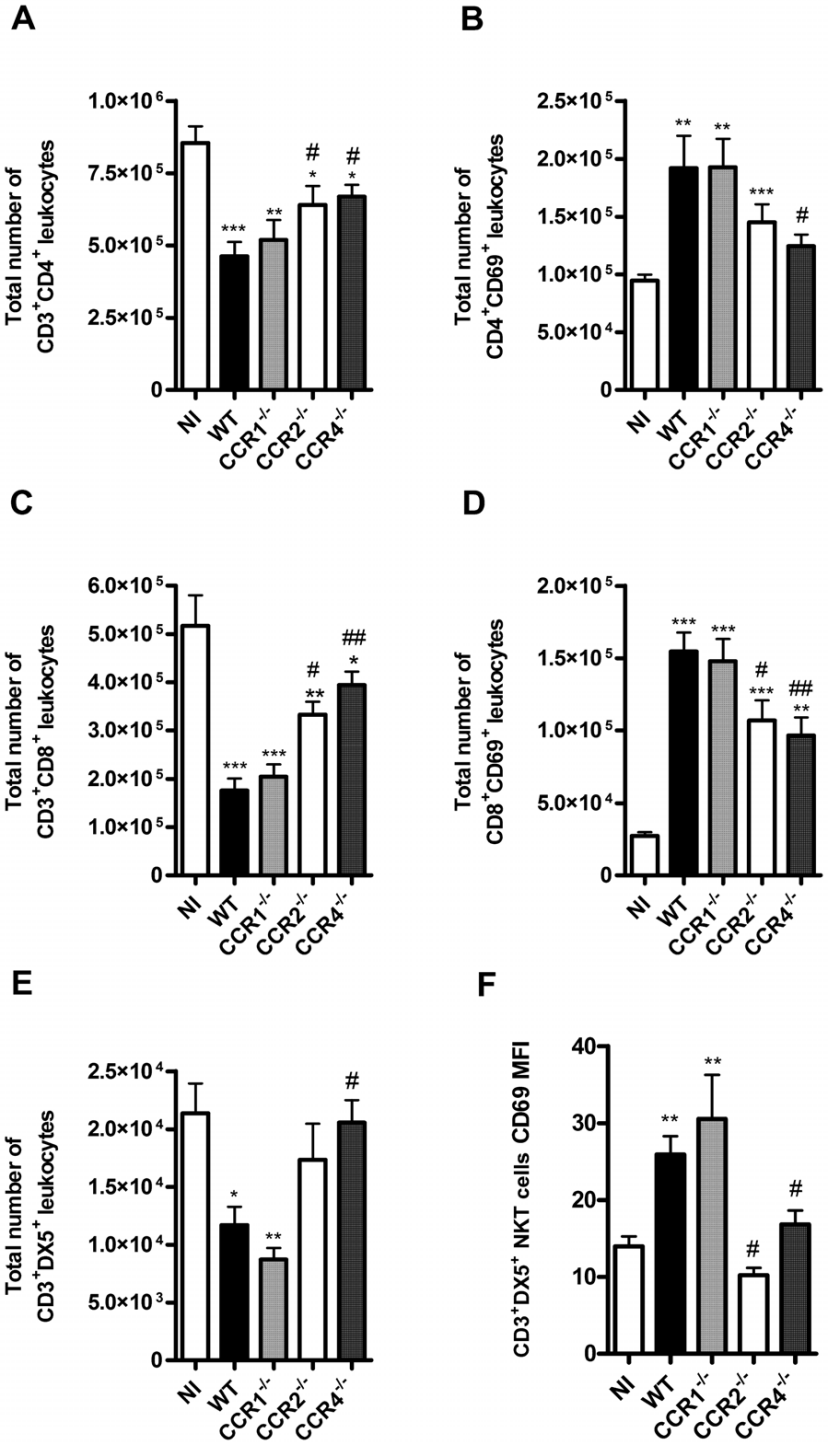
Lymphocyte number and activation in CC chemokine receptors deficient mice upon DENV-2 infection. WT or KO mice were infected i.p. with 10 LD_50_ of DENV-2 and then sacrificed at day 6. Splenic leukocytes were counted and then stained with specific antibodies. Flow cytometry, according to size and granularity, were performed as analysis. The numbers of specific cell populations are shown compared to total number of leukocytes in the spleen. Number of T lymphocytes CD3^+^CD4^+^ (A) and CD3^+^CD8^+^ (C) were evaluated in WT and KO mice. Activated T lymphocytes expressing CD69 were also evaluated for CD4^+^ (B) and CD8^+^ (D) populations. The number of CD3^+^DX5^+^ NKT cells (E) and their activation by CD69 expression as MFI, were also evaluated (F). Results are expressed as mean ± SEM and are representative of at least two experiments (n = 5–6 mice). *P<0.05, ** P<0.01, *** P<0.001 when compared to non-infected mice. # P<0.05, ## P<0.01 when compared to WT infected mice. NI: non-infected mice. MFI: Mean fluorescence intensity.

Next, we evaluated the number of leukocytes in spleen of WT and chemokine receptor deficient mice in the course of dengue infection. Overall, DENV-2 infection caused significant reduction in the total number of CD3^+^CD4^+^, CD3^+^CD8^+^ and NKT cells (CD3^+^DX5^+^) in the spleen of infected when compared to non-infected WT mice (Figure 7A, C and E). Despite reduction in total cell numbers, activated CD8^+^, CD4^+^ T and NKT cells, as determined by CD69 expression, were more abundant in spleen of infected mice (Figure 7B, D and F). Similar findings were observed in CCR1^–/–^ mice; ie. decreased number of cells but available cells were more activated (Figure 7). In contrast, absence of CCR2 or CCR4 partially reversed the phenotype observed in WT mice. Indeed, there was a lesser decrease in total CD8^+^, CD4^+^ T and NKT number and cells tended to be less activated (Figure 7).

**Figure 8:**
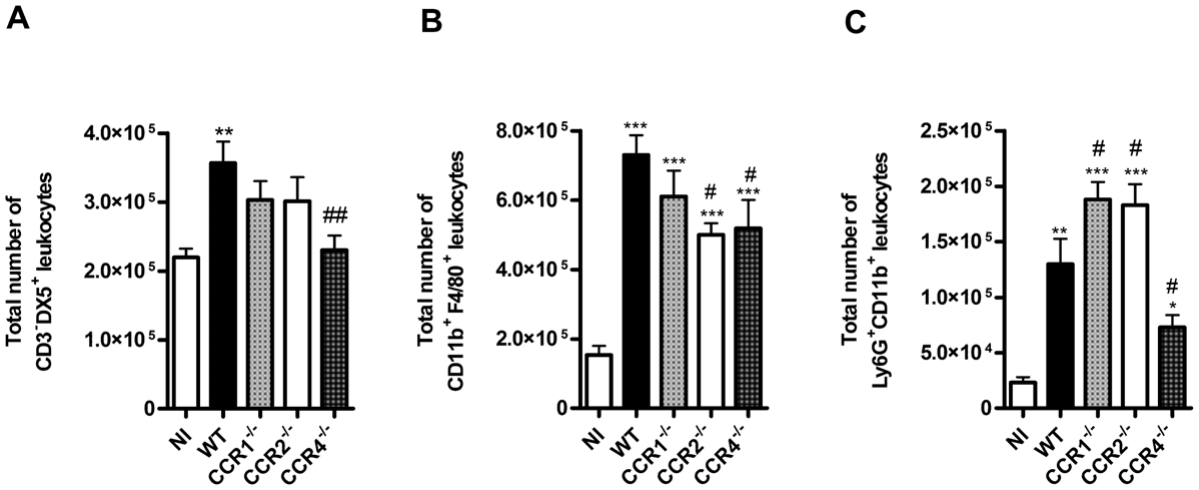
NK, macrophages and neutrophils number in CC chemokine receptors deficient mice upon DENV-2 infection. WT or KO mice were infected i.p. with 10 LD_50_ of DENV-2 and then sacrificed at day 6. Splenic leukocytes were counted and then stained with specific antibodies. Flow cytometry, according to size and granularity, were performed as analysis. The numbers of specific cell populations are shown compared to total number of leukocytes in the spleen. Numbers of CD3^−^DX5^+^ NK cells (A), macrophages CD11b^+^F4/80^+^ (B) and infiltrating neutrophils CD11b^+^Ly6G^+^ (C) were evaluated. Results are expressed as mean ± SEM and are representative of at least two experiments (n = 5–6 mice). *P<0.05, ** P<0.01, *** P<0.001 when compared to non-infected mice. # P<0.05, ## P<0.01 when compared to WT infected mice. NI: non-infected mice.

In contrast to the CD8^+^, CD4^+^ T and NKT lymphopenia, DENV-2 infection caused significant increase in the number of NK cells (CD3^–^DX5^+^), macrophages (CD11b^+^F4/80^+^) and neutrophils (CD11b^+^Ly6G^+^) (Figure 8A–C). Again, infected CCR1^–/–^ mice were similar to infected WT mice, with the exception of an increase in number of neutrophils in spleen. CCR2^–/–^ mice had only mild changes in comparison to their WT controls, whereas CCR4^–/–^ mice had decreased accumulation of all 3 cell types in spleen (Figure 8).

## Discussion

In the present work we have investigated the putative role of CC chemokine receptors CCR1, CCR2 and CCR4 in the context of experimental *Dengue virus* infection. Our major findings can be summarized as follows: 1) CCR1 does not seem to have a major role in the pathogenesis of severe experimental dengue infection; 2) CCR2 appeared to contribute to dengue-associated liver damage and this was reflected on decreased leukocyte activation and decreased lethality. However, there was no major difference in the systemic inflammatory response associated with infection; 3) CCR4 also contributed to the pathogenesis of experimental dengue infection and was relevant for virus-induced liver damage and associated systemic inflammation. This was reflected on the decreased leukocyte activation and decreased lethality.

CCR1 receptors are widely expressed in monocytic and non-monocytic populations [29], [30], [31]. CCR1 ligands, such as CCL3/MIP-1α and CCL4/MIP-1β, are found in elevated concentrations in plasma of patients with DENV infection and may be associated with disease severity [37], [38], [39]. Consistent with the latter finding, levels of CCL3/MIP-1α are increased in spleen and liver of infected mice. However, we found that the course of infection in CCR1^–/–^ was similar to that in WT mice. Levels of CCL3 were greater in spleen and liver of infected CCR1^–/–^ than infected WT animals. This is in agreement with the idea that chemokine receptors work as important negative modulators or scavengers of their own ligands and lower their levels in tissues [58]. In that respect, elevated levels of CCL3 could then activate the other CCL3 receptor, CCR5. We have not investigated here the role of CCR5 to infection outcome but it is clear that CCR1^–/–^ mice had no major phenotype when infected with an inoculum which causes severe disease in mice. Therefore, CCR1 does not appear to play a major role in the pathogenesis of severe experimental dengue infection.

CCL2/MCP-1 is produced under many inflammatory conditions and exerts its functions mainly through the CCR2 receptor, which is expressed in monocytes, macrophages and also neutrophils [25], [27], [31], [59]. Levels of CCL2 have been positively associated with worse prognosis in DENV-infected subjects [21], [37]. In our experiments, survival rates were reduced in CCR2^–/–^ mice and this was associated to decreased levels of serum transaminases and increased liver inflammation and damage. Of interest, hepatocytes are common targets of DENV and can actively respond to the infection by producing cytokines and chemokines [41]. The degree of thrombocytopenia and hemoconcentration was similar in WT and CCR2^–/–^ mice, but levels of IL-6 and IFN-γ, but not TNF-α, were decreased systemically in infected CCR2^–/–^ mice. There was also decreased activation of major cell types involved in DENV infection, including CD4+, CD8+, NKT cells and macrophages. Interestingly, preliminary data from our group shows that NKT cells contribute to the “cytokine storm” associated with DENV infection in mice (Renneson *et al*., unpublished data). Therefore, decreased leukocyte activation in infected CCR2^–/–^ mice may explain the decreased cytokine storm and decreased tissue damage observed in these animals. Increased plasma extravasation is thought to lead to hemoconcentration, hypotension and death [21], [50], [51], [52], [53]. It is, therefore, not clear why there is still significant hemoconcentration in CCR2^–/–^ mice at day 6 after infection, despite decreased cytokine levels, liver damage and lethality rates. It is possible that a few animals may eventually recover from the massive vascular permeability which leads to hemoconcentration because they have more adequate liver function. However, these are difficult experiments to perform because control animals are mostly dead at later stages of infection and there would be few control animals with which to compare the CCR2^–/–^ mice. Surviving CCR2^–/–^mice have normal blood parameters at later stages (day 14) of infection (data not shown). Thus, CCR2 plays a role in the pathogenesis of severe experimental dengue infection and it appears that enhanced survival in CCR2^–/–^ mice is probably secondary to decreased liver damage, decreased cell activation and decreased cytokine storm.

The CCR4 receptor is expressed on T cells, especially Th2-type lymphocytes, and may contribute to the pathogenesis of severe conditions, including asthma [60], [61], [62], [63], [64], [65]. Interestingly, CCR4 deficiency results in attenuated severity of murine polymicrobial sepsis and lipopolysaccharide-induced endotoxic shock, implicating this receptor in the pathogenesis of acute conditions [66], [67]. Other experiments, however, have found a protective role for CCL22/MDC, a CCR4 ligand, in a cecal ligation and puncture (CLP) model of sepsis in mice [68]. In preliminary experiments, we found that CCL17/TARC, one of the ligands for CCR4, was detectable at high levels in spleen of infected mice. More importantly, experiments in CCR4^–/–^ showed that these animals were protected from DENV-associated disease. Indeed, there was decreased hemoconcentration, thrombocytopenia, liver damage, systemic inflammation and leukocyte activation in CCR4^–/–^ mice. This resulted in significant protection from lethality. These results imply a crucial role of this receptor in the pathogenesis of DENV-associated severe disease. Importantly, viral load was not altered in CCR4-/- when compared to WT animals. These results suggest that CCR4 does not play a major role in the control of viral entry and replication, but contribute mostly to the cascade of events that lead to tissue and systemic damage. In this respect, our findings are consistent with the protective role of CCR4 in the pathogenesis of bacterial sepsis [66], [67]. It is difficult to suggest the mechanism by which CCR4 may contribute to the pathogenesis of dengue. However, CCR4 may be important for the trafficking and activation of NKT cells and naive CD8+ cells by at least two independent chemokine pathways, including CCL17/TARC and CCL22 [69], [70]. In addition, excessive NKT activation contributes to the pathogenesis of severe disease in our model (Renneson *et al*., unpublished data). Thus, at least in viral (present study) and polymicrobial sepsis, blockade of CCR4 may be beneficial from the therapeutic point of view, a tenet that must be tested further in patients. Indeed, we are unaware of studies demonstrating the putative role of CCR4 ligands, such as CCL17 and CCL22, in the context of DENV infection in humans. In addition to the present findings, the protection observed in CCR2^–/–^ and CCR4^–/–^ mice upon DENV-2 infection could be further explored with strategies such as using double knock-out mice for these receptors, which could bring a more concise picture on the role of these receptors in the inflammatory response and shock associated syndrome observed in dengue. Furthermore, future studies should investigate the role of chemokines in mediating humoral and cellular responses against the pathogen, mostly in order to understand whether and how chemokines regulate lymphocyte trafficking in the context of dengue.

In conclusion, CCR1, CCR2 and CCR4 play discrete roles in the pathogenesis of disease in a model of DENV in mice. In contrast, these receptors appear not to play an essential role in protection against primary infection. Our studies suggest that the chemokine storm that follows severe primary dengue infection associates mostly to development of disease rather than protection against severe infection. It is, therefore, possible that blockade of the chemokine system may be beneficial as co-adjuvant treatment for severe dengue infection.
